# List of Accidents, Admitted into the Pennsylvania Hospital, from July 25th to August 8th, 1838

**Published:** 1838-08-15

**Authors:** 


*List of Accidents, admitted into the Pennsylvania Hospital, from July 25th to August 8th*, 1838.

One case of fracture of both bones of the leg, from a fall from a waggon; treated by a fracture-box, with cloths of lead water.—One case of compound fracture of the parietal bone, without depression, caused by the fall of an axe from the third story of a house: the patient has been very freely depleted, and salivated by two purgative doses of calomel; now doing well.—One case of lacerated wound of the head, with fracture of the outer table of the bone: the wound was drawn together with adhesive straps.—One case of lacerated wound of the head—dressed in the same manner.—One case of *comminuted fracture of the os calcis*, caused by a fall upon the feet from a third story window, in a fit of intoxication, accompanied by a lacerated wound of the same foot, which did not, however, communicate with the fracture. The wound mortified, and, subsequently, extensive mortification took place in the loins. The patient was treated with brandy, carbonate of ammonia, camphor and opium, but died on the fifth day from the occurrence of the injury. This case is interesting, from the exceeding infrequency of the accident. One case of fracture of the os calcis is mentioned by Baron Boyer, observed in the hospital de la Salpétrierè, and one by Desault, at the Hôtel-Dieu, both of which were produced by the same causes, as the present,—direct violence, from a fall on the heel, from a considerable height. The accident was recognised, in this instance, by a very distinct crepitus, but there was no displacement, and "the extremity of the bone" was not "found separated from its body and drawn upward on the posterior surface of the leg, forming a distinct tumour," by which sign the nature of the case is manifested, according to Professor Gibson, (*Surgery*, 4th ed., vol. i. p. 303.) The seat of fracture, determined by dissection after death, was beneath and somewhat anterior to the articulation with the astragalus, contrary to Boyer's idea that "fracture of the os calcis always takes place in the part of this bone, comprised between its articulation with the astragalus and its posterior extremity." (*Traité des Maladies Chirurgicales*, tome iii., p. 389.) The bandage, usually recommended for the treatment of fracture of the os calcis, would, in this case, have been, of course, unnecessary.—One case of sprain of the knee: treated by leeching, rest, and cold applications.—One case of fracture of the lower third of the thigh, from a fall, in a woman, eighty years of age: treated with a double inclined plane of pillows.—One case of contusion of the abdomen, from a blow with a large block of anthracite coal: treated by dry cupping and poultices of flax seed, with the tincture of valerian and laudanum, to meet symptoms of incipient mania a potu.—One case of contusion of the hip, in a child eight years of age, produced by extension, made in an attempt to reduce a supposed dislocation of the head of the thigh-bone. The child was brought to the hospital, for the application of the pulleys to effect the reduction of what was stated to be a dislocation of the head of the thigh-bone into the ischiatic notch, and which had been attempted by extension and counter-extension, with venesection, and the internal exhibition of tartarized antimony. The bone had been similarly luxated, it was said, a week previous, and reduced. The condition of the limb, which had subjected the child to so much unavailing suffering, was the following, constituting a case of incipient coxalgia: the length of both limbs, from the anterior superior spinous processes to the internal malleoli, was *the same;* a depression of the right side of the pelvis, of about three quarters of an inch from lateral curvature of the spine, occasioned an *apparent* elongation of the corresponding limb, which the child threw somewhat forward, when in the erect posture, resting his weight mainly on the sound limb. The depression of the right side of the pelvis occasioned a corresponding depression of the trochanter, and the wasting of the muscles of the same side gave it the appearance of unnatural projection, as well as of displacement. At the time of the boy's admission, he would not permit free rotation of the limb, owing to the condition of his muscles, from the severe extension he had undergone. The succeeding day, however, after rest, and the application of a few leeches to the groin, perfect rotation and flexion of the limb could be practised. The boy is now under treatment for coxalgia.—One case of fracture of the neck of the humerus, in a man ninety years of age: treated with the clavicle apparatus, without a pad.

The incised wound of the arm, and of the wrist, mentioned in the last number, have been discharged, cured. The cases of mania a potu, spoken of in the same number, have been cured, and the patients are now under treatment for the fractures. The contused wound of the thigh, made by the bar of red-hot iron, has been discharged, cured.

